# A Chatbot-Based Version of the World Health Organization–Validated Self-Help Plus Intervention for Stress Management: Co-Design and Usability Testing

**DOI:** 10.2196/64614

**Published:** 2024-10-18

**Authors:** Valentina Fietta, Silvia Rizzi, Chiara De Luca, Lorenzo Gios, Maria Chiara Pavesi, Silvia Gabrielli, Merylin Monaro, Stefano Forti

**Affiliations:** 1 Fondazione Bruno Kessler Trento Italy; 2 Department of General Psychology University of Padova Padova Italy; 3 Istituto Pavoniano Artigianelli Trento Italy

**Keywords:** acceptance and commitment therapy, ACT, well-being, pregnancy, breast cancer, eHealth, mobile health, mHealth, development, usability, user-centered design

## Abstract

**Background:**

Advancements in technology offer new opportunities to support vulnerable populations, such as pregnant women and women diagnosed with breast cancer, during physiologically and psychologically stressful periods.

**Objective:**

This study aims to adapt and co-design the World Health Organization’s Self-Help Plus intervention into a mobile health intervention for these target groups.

**Methods:**

On the basis of the Obesity-Related Behavioral Intervention Trials and Center for eHealth Research and Disease Management models, low-fidelity and high-fidelity prototypes were developed. Prototypes were evaluated by 13 domain experts from diverse sectors and 15 participants from the target groups to assess usability, attractiveness, and functionality through semantic differential scales, the User Version of the Mobile Application Rating Scale questionnaire, and semistructured interviews.

**Results:**

Feedback from participants indicated positive perceptions of the mobile health intervention, highlighting its ease of use, appropriate language, and attractive multimedia content. Areas identified for improvement included enhancing user engagement through reminders, monitoring features, and increased personalization. The quality of the content and adherence to initial protocols were positively evaluated.

**Conclusions:**

This research provides valuable insights for future studies aiming to enhance the usability, efficacy, and effectiveness of the app, suggesting the potential role of a chatbot-delivered Self-Help Plus intervention as a supportive tool for pregnant women and women with a breast cancer diagnosis.

## Introduction

### Background

The growing awareness of the profound significance of mental health for individuals and society has spurred an expanding body of research to scrutinize global population trends and the strategies used to address this issue. Empirical evidence consistently reveals an enduring surge in requests for psychological support, yet this burgeoning demand remains largely unmet due to scarce available resources and services [1]. Consequently, people’s needs remain unmet. The factors contributing to the challenge of accessing mental health services are multifaceted. In general, these impediments encompass issues such as suboptimal service quality, inadequate levels of mental health literacy, pervasive stigma, and formidable cost barriers [2]. Within this context, developing and implementing strategies to fortify and enhance the health care system becomes increasingly imperative, rendering it more accessible to the population. Significantly, particular attention is being devoted to the prospective role of digital technologies, which can enhance the sustainability of the health care system by providing 24/7 support to patients and optimizing health care provider interventions [3]. These innovations aim to surmount the aforementioned impediments, advancing digital health as an integral and foundational strategy to foster equitable, affordable, and universally accessible mental health care [4]. A flourishing body of literature corroborates the potential of emergent technologies, encompassing telemedicine, mobile health (mHealth) initiatives, and digital therapies, in facilitating a seamless continuum of care, extending from clinical settings to patients’ homes while embracing a staged care approach [3].

Furthermore, digital health may be particularly suitable for low-intensity mental health interventions [5]. This terminology refers to specific programs wherein the active engagement of health care professionals and specialists is not necessarily required. These interventions are grounded in empirically validated [6], evidence-based psychological practices seamlessly integrated into a self-help paradigm, whether guided or unguided. This intervention genre is conventionally designed to be transdiagnostic, offers facile adaptability across diverse contexts, and is readily implementable by nonprofessional operators. Given their structural attributes and overarching mission, low-intensity interventions represent a valuable conduit for augmenting access to pragmatic, evidence-based psychological treatments, catering to a broad spectrum of recipients. These encompass from individuals among the general population to those presenting with limited or mild symptomatic manifestations associated with distress and mental illness [6]. In summary, low-intensity interventions are positioned as a pivotal resource for addressing situations characterized by mild distress, in which failure to intervene effectively could potentially precipitate the escalation of these conditions into pathological states [7].

Psychological distress, encompassing stress, anxiety, and depression, frequently co-occurs with physical illnesses such as breast cancer [8]. Cultivating a more optimistic outlook has been demonstrated to play a role in disease management and recovery, underscoring the significance of holistically addressing physical conditions and mental health issues [9,10].

Even a completely different health condition from those mentioned previously, such as pregnancy, can expose women to similar psychological symptoms. Pregnancy is characterized by major transformations that significantly impact the woman physically, mentally, and socially. How the woman adapts to these changes determines the quality of her life and her levels of well-being [11]. Where adaptation is not functional, symptoms of psychological distress may occur; the most common conditions are anxiety, stress, and depression [11-14]. To date, psychoeducational interventions that promote women’s psychological well-being during pregnancy are scarce and tend to focus mainly on samples of women with psychiatric symptomatology (eg, perinatal depression disorder) [15]. For this reason, our study is part of the digital health framework to support health prevention strategies in the first 1000 days of life [16].

Evidence of the effectiveness of psychological interventions targeting pregnant women or women with breast cancer is increasing [17,18], but access to care services still presents several challenges. Many women face geographic barriers, with specialized centers often far from their homes. In addition, a shortage of qualified personnel, such as psychologists, further limits access to specialized care [19]. The stigma associated with mental health problems can also prevent women from seeking psychological support [20]. Therefore, in numerous instances, these target groups are excluded from accessing the requisite services. However, low-intensity interventions emerge as pivotal in addressing this lacuna, and the strategic combination with mHealth methodologies can yield an effective, sustainable, and inclusive framework for augmenting the scalability of mental health interventions.

This model is poised to cater to a comprehensive user base, encompassing prevention for individuals at potential risk of mental distress and intervention for patients grappling with mild to moderate mental distress [7].

### Self-Help Plus

Self-Help Plus (SH+) is a low-intensity group intervention for stress management initially developed to target populations that are numerous or hard to reach by health care professionals under the principle of improving and facilitating access to health care interventions [21]. The SH+ package has been incorporated into the expanding array of low-intensity psychological interventions endorsed by the World Health Organization (WHO) [21]. By design, SH+ is a transdiagnostic intervention that is applicable, meaningful, and safe for people with and without mental disorders. SH+ is based on acceptance and commitment therapy (ACT) [22,23], a form of cognitive behavioral therapy [24,25].

The SH+ intervention package has 3 main components: a prerecorded audio course, a facilitator manual, and a self‐help booklet for participants. This material has been translated into multiple languages and can be easily accessed on the web at the WHO website [26]. The audio material imparts key information about stress management and guides participants through individual exercises and small group discussions. The intervention is structured into five sessions focused on acceptance- and mindfulness-based techniques for stress management: (1) *grounding* (mindfulness), (2) *unhooking* (cognitive distancing), (3) *acting on your values* (value-based behavioral activation), (4) *being kind* (gratitude), and (5) *making room* (acceptance).

Preliminary studies report positive effects of SH+ with a potential impact on mental well-being; it had a significant long-term efficacy in a target population of refugees and asylum seekers exposed to stressful situations [27]. Other studies show a still debatable effect of SH+ when applied to health care professionals during the COVID-19 pandemic, whereas there emerges a need for further examining the potential role of the confounding effects of nonspecific factors [28].

### This Research

This research fits into the landscape of WHO strategies by adapting the stress management intervention developed by the WHO itself, SH+, with two main goals: (1) to assess the viability of this intervention when targeting specific subgroups (women with breast cancer and pregnant women) and (2) to validate the applicability of the intervention as a chatbot-delivered and preventive action. This SH+ intervention, which has already been validated and tested on some specific vulnerable populations (eg, asylum seekers) [29], will be fully available to users through digital tools. In particular, it will be delivered through a mobile app and guided by a virtual assistant, ALBA. This research aims to assess the prototype of the ALBA app to gather feedback and needs from key stakeholders to further refine the app from a qualitative perspective of usability, accessibility, and acceptability of the intervention delivered via a chatbot.

## Methods

### Overview

**Figure 1:**
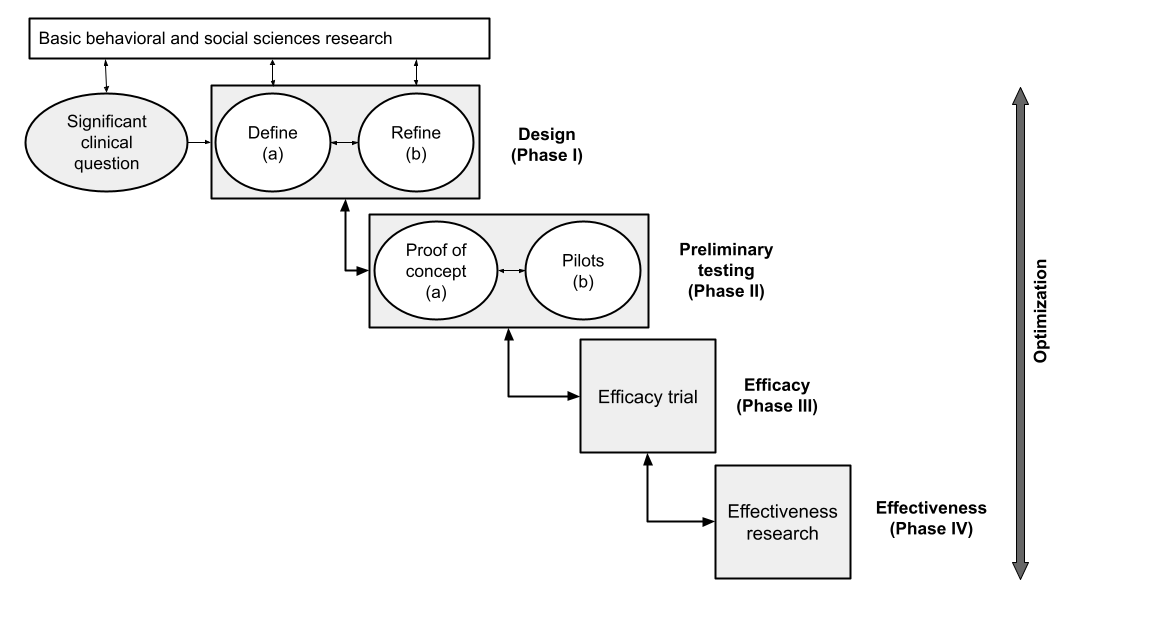
The Obesity-Related Behavioral Intervention Trials model.

The stress management intervention was developed iteratively following the Obesity-Related Behavioral Intervention Trials (ORBIT) model [30], as illustrated in Figure 1 [30], which shows the pathway followed to translate a human-guided intervention into a possible digital therapy. In particular, the design and development process encompassed a multidisciplinary approach and continuous, systematic evaluation throughout, as the Center for eHealth Research and Disease Management (CeHRes) comprehensive road map approach recommended to improve the uptake and impact of eHealth technologies [31].

The multidisciplinary project team, consisting of psychology, eHealth research, and communication experts, had biweekly meetings during the design and development phase. User-centered design methodologies ensured user involvement throughout the design and development process. Patient representatives, health care providers, and security experts were consulted throughout. The stress management intervention was developed in iterative processes through a combination of (1) intervention content development, identified and adjusted from the evidence-based cognitive behavioral stress management concept; and (2) iterative software development (phase 1—low-fidelity prototypes and phase 2—high-fidelity prototypes) and formative evaluation.

### Intervention Content Development

A primary goal of this study was to adapt a validated stress management intervention, SH+ [21], into a new technology-based stress management intervention for pregnant women and women with breast cancer. To do so, the development involved a wide range of expertise encompassing psychology, eHealth IT, interaction design, and specialized knowledge in pregnancy and oncology. A multidisciplinary team was assembled to address diverse user needs, ensure psychological coherence, and integrate technological requirements and user-centered design principles. The focus was on achieving adaptability of the proposed tools in the digital domain [31] guided by 2 pivotal methodologies: user-centered design [32,33] and service design [34].

The development process comprised the following stages: (1) literature review of WHO protocols and papers on digital mental health and the specific psychological needs of target populations; (2) exploration gathering insights from user representatives (eg, patients with breast cancer and pregnant women), health care providers, and eHealth experts (including designers and developers); and (3) content adaptation customizing SH+ intervention manual content addressing software development and potential privacy and security issues. The intervention content was adapted and tailored by the entire research team through iterative processes to fit a 5 module–based intervention in electronic format. Adjustments were made to ensure easy language, short sentences, and focus on clear content for small screens.

This holistic approach, merging diverse expertise and user-centric methodologies, underscores the dedication to crafting a robust and efficient chatbot-driven intervention tailored for women dealing with breast cancer and pregnancy.

### Phase 1: Iterative Development and Low-Fidelity Prototypes

The adaptation of the SH+ intervention through the implementation of the ALBA chatbot was based on a novel approach to delivering psychological support. Users can engage with a comprehensive and effective intervention through gamification, personalized sessions, reminders, and feedback. Therefore, a key aspect is represented by a multilevel structure to consider the different sections of the app and, simultaneously, to guarantee proper levels of user engagement, adherence, and overall impact on users’ well-being. In the first iteration in 2023, a total of 3 psychologists and 2 communication experts tested and gave feedback on the prototype to ensure that the intervention program was logically built and would meet the stakeholder requirements.

On this basis, the following methodology was applied. The group of experts was divided into pairs, in which one person assumed the role of the chatbot whereas the other adopted the user’s perspective, reading their respective segments of the dialogue aloud.

The pairs were reorganized for each of the 5 distinct modules to ensure a diverse spectrum of interactions and exhaustive coverage of potential dialogue scenarios and to avoid biases. This method facilitated the exploration of varied interaction dynamics and the collection of data on multiple communication styles. The oral recitation of dialogues served as a mechanism to evaluate several aspects of the chatbot’s effectiveness, such as dialogue realism, rhythmicity and repetition of the texts, and communication fluency [35]. The verbalization process aids in gauging how seamlessly the chatbot replicates a humanlike conversation, identifying any inconsistencies or unnatural responses. Thus, listening to the dialogue’s progression allows for the assessment of the conversation’s smoothness, which encompasses the coherence and pertinence of the chatbot’s replies. Another function of this method was to identify any ambiguities or misinterpretations that might emerge during interactions with the chatbot [35]. Simulating the conversation enabled the experts to offer immediate critiques on every facet of the dialogue, contributing to rapid and focused content refinement.

In a nutshell, this procedure aimed to analyze the chatbot’s capability to engage in realistic, empathetic, and psychologically suitable conversations, leveraging direct feedback and expert psychologists’ insights as key metrics for evaluation.

### Phase 2: Iterative Development and High-Fidelity Prototypes

After minor adjustments, the paper prototype was implemented into an electronic format using the Landbot tool (HELLO UMI S.L.) [36]. Landbot is a chatbot generator that allows one to create, test, and deploy conversational chatbots via WhatsApp and other chat channels.

**Figure 2:**
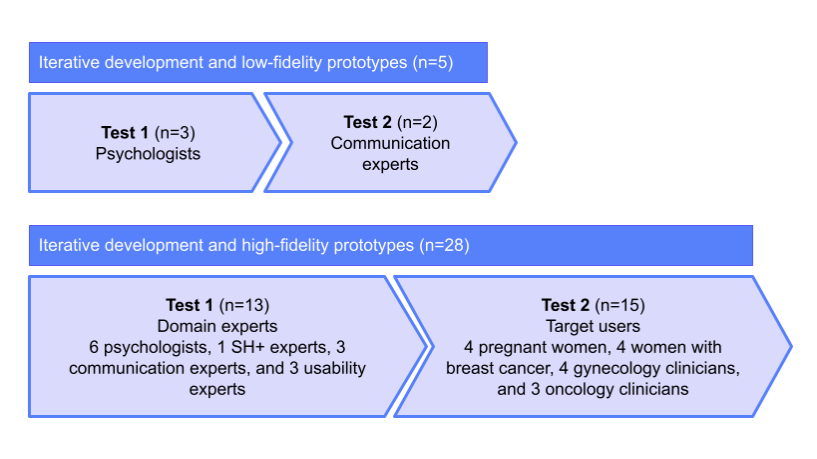
Software development and formative evaluation (N=37). SH+: Self-Help Plus.

During the period spanning the end of 2023 and the beginning of 2024, a 2-phase evaluation of the ALBA prototype was conducted involving an overall sample of 28 participants, as elaborated on in Figure 2. In particular, of the 28 participants involved, 13 (46%) were domain experts (n=6, 21% psychologists), 1 (4%) was an SH+ expert, 3 (11%) were communication experts, 3 (11%) were usability experts, and 15 (54%) were users—8 (29%) target users (n=4, 50% women who were currently pregnant or had given birth within the previous year, 4/28, 14% of the total sample; and n=4, 50% women who were in current breast cancer disease status or follow-up from it, 4/28, 14% of the total sample) and 7 (25%) target clinicians (n=4, 57% gynecology clinicians [eg, obstetricians and gynecologists; 4/28, 14% of the total sample] and n=3, 43% oncology clinicians [eg, oncologists and case managers; 3/28, 11% of the total sample]). The recruitment of participants was based on personal contacts of researchers selected based on representativeness of the key target user groups addressed by the ALBA solution.

### Variable Identification

In an attempt to gather the necessary information, key variables were identified for investigation: communication, session structure, materials, engagement, functionality, esthetics, information, subjective and perceived impact, interaction, communication mode, involvement consistency, general comments, amelioration of technical implementation, and content adherence. The first 4 variables were assessed through the semantic differential tool [37], the following 5 were assessed through the User Version of the Mobile Application Rating Scale (uMARS) [38], and the latter 6 were assessed through an ad hoc semistructured interview.

The semantic differential tool is an instrument consisting of a series of scales, each of which is composed of a pair of bipolar adjectives between which a rating scale (5 positions) is placed. Given the study’s variables, a list of subvariables was chosen to create ad hoc items for the research. Table 1 shows the chosen variables and their respective subvariables. On the basis of the target subject domain, each person was asked to evaluate and determine variables, as reported in Table 1 (single items are reported in Multimedia Appendix 1).

**Table 1:** List of variables investigated through the semantic differential tool and the people involved in evaluating the individual variables.

Variable and subvariable			Psychologists		SH+^a^ expert		Communication experts		Usability experts		Target group		Clinicians
**Communication**													
	Empathy and listening	✓^b^		✓		✓				✓		✓	
	Smoothness and fluidity	✓		✓		✓		✓		✓		✓	
	Chatbot interaction	✓		✓		✓		✓		✓		✓	
	Lexicon	✓		✓		✓		✓		✓		✓	
**Session structure**													
	Interaction length	✓		✓		✓		✓		✓		✓	
**Materials**													
	Audio tracks	✓		✓		✓				✓		✓	
	Infographics and videos	✓		✓		✓				✓		✓	

^a^SH+: Self-Help Plus.

^b^Involved.

The uMARS questionnaire, on the other hand, evaluates mobile apps by covering 4 objective dimensions (engagement, functionality, esthetics, and information) and 1 subjective dimension. Briefly, the questionnaire consists of 20 items covering the inquired variables as follows—engagement (n=5, 25%), functionality (n=4, 20%), esthetics (n=3, 15%), and information (n=4, 20%)—and 4 items belonging to the subjective quality domain. A section on perceived impact (6 items) also assesses users’ perceptions of the app’s usefulness. Each answer is rated on a 5-point scale (1=inadequate, 2=poor, 3=acceptable, 4=good, and 5=excellent) measuring the usability of mHealth apps.

Interviews, instead, are suitable for a more in-depth investigation of users’ attitudes and preferences regarding new technological solutions as open-ended discussions with users can help researchers better understand the issues and concerns related to the possible future adoption of these solutions [39]. Multimedia Appendix 2 shows the list of topics and questions posed during the interviews.

### Context-Specific Methodologies

#### Personas

**Figure 3:**
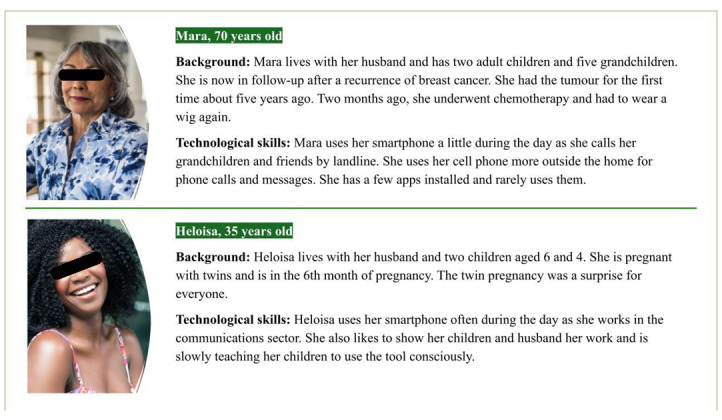
Examples of personas.

Personas are a user-centered and service design method used to create and visualize fictional representations of the target group [40]. Personas are an effective method for all project team members to better understand the target group for which the app is built. Personas in this study contained information about the pregnancy background, challenges, and technology use. Figure 3 provides illustrated examples of study personas. Psychologists had to take the perspective of one of these personas before the reading.

#### Focus on Experience and Expertise

This study aimed to involve experts from various fields and did not require all participants to respond to every specific ad hoc item of the semantic differential tool and questions of the semistructured interview. Usability experts were not asked to evaluate specific intervention content, whereas the SH+ expert was queried about the fidelity of the content to the original intervention during the interview. Clinicians, psychologists, and participants from the pregnancy context were asked questions relevant to that context, whereas those from the oncology field responded to questions specific to their area of work or direct life experience.

### Procedure

All data were collected confidentially with participants’ informed consent. Recruitment was conducted through word of mouth and direct acquaintance, clearly stating the study’s objectives. Only volunteers aged ≥18 years were included. Before the study, participants received a privacy notice and consent form via Google Forms, allowing them to consent to participation and data processing.

**Figure 4:**
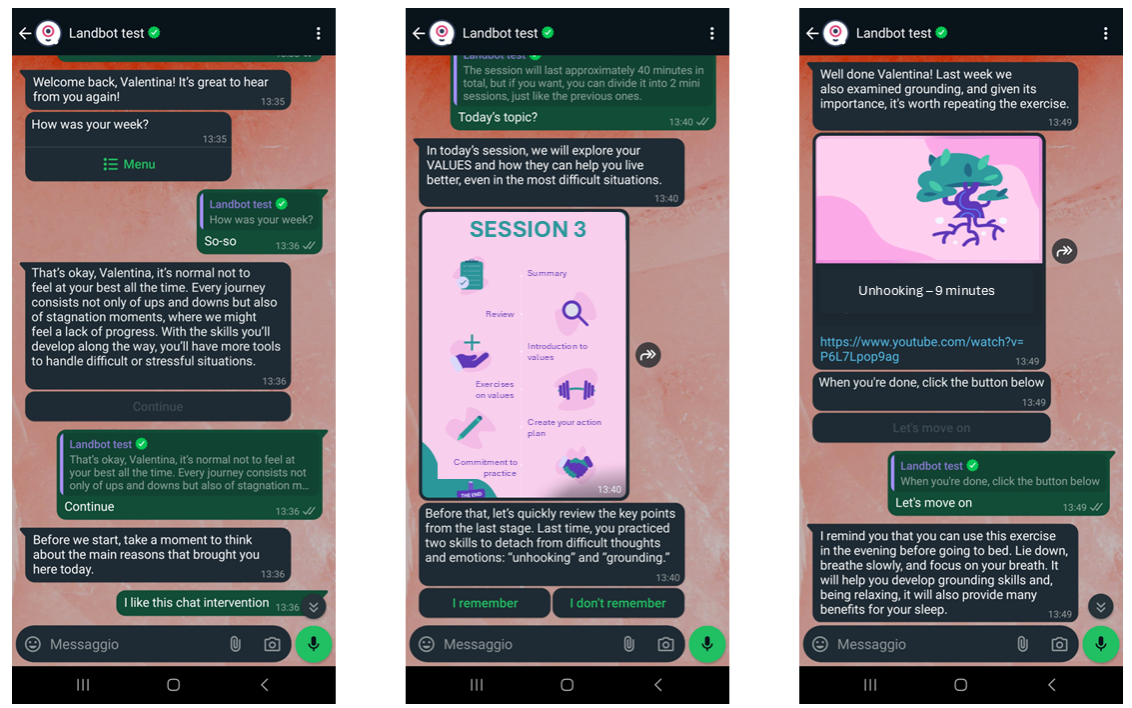
Examples of WhatsApp chat mock-ups. ALBA delivers content and monitors users’ experiences by assigning tasks and providing motivational feedback. Through ALBA, users can interact, receive clear instructions, and access exercises and multimedia content.

Operationally, the experimental procedure was structured as follows:

1. The information notice and informed consent were displayed.
2. Participants who decided to take part in the study were asked to fill out a questionnaire that collected some generic biographical information (age, schooling, gender, and employment status) and some information related to their knowledge and use of mobile apps.
3. Next, participants interacted with the ALBA app prototype for 6 weeks. Each session lasted approximately 40 minutes. During the study, the participants were asked to test the ALBA app on their phones; in particular, they had the opportunity to explore the interface and different sections of the app via mock-ups and read, listen to, and interact with the chatbot via WhatsApp during the dialogue session (see Figure 4 for ALBA chat mock-ups). The session consisted of structured dialogues with predefined buttons for response options or free text. The text was not analyzed in this prototype phase as it was not the main purpose of this study. The dialogue was constructed to be as standardized as possible to maintain the already tested validity of the intervention. Participants were also informed about the future implementation of reminders and feedback for the activities proposed by the chatbot.
4. Questionnaires were then presented to the participants regarding their overall experience with the system. In the final stages, participants were asked to evaluate the experience they had with the ALBA app by answering 2 questionnaires. Specifically, the semantic differential tool [37] and the Italian version of the uMARS [41] were used for the evaluation through questionnaires.
5. Finally, a brief semistructured interview was conducted. After the usability assessment, participants were invited to join a web-based interview to further report on their expectations, preferences, and concerns regarding the ALBA solution tested. The interview questions were specifically designed ad hoc for our study, focusing on topics relevant to the evaluation of the app, such as interaction, communication mode, user involvement and consistency, technical implementation, and content adherence. The inclusion criteria for participating in the interview were having completed both the prototype test and the questionnaires as well as providing consent to participate in this additional phase. The exclusion criteria, on the other hand, were having dropped out during the test, not completing the questionnaires, or not providing consent for the interview. A total of 28 interviews were conducted by a researcher and audio recorded to enable a more detailed analysis of participants’ responses. They were then analyzed and processed using qualitative tools. The interviewer initially provided a brief introduction to the interview objectives. Then, participants were asked to answer a series of semistructured questions regarding their expectations and preferences for using the app.

To sum up, this study was divided into 2 main phases, each with distinct timelines and activities. Phase 1 focused on the iterative development of low-fidelity prototypes and spanned 6 months. Phase 2, dedicated to the iterative development of high-fidelity prototypes, lasted 4 months. Each step of phase 2 was carefully structured with specific time frames to ensure thorough data collection and analysis—10 minutes for informed consent completion, 15 minutes for pretest questionnaire completion, 6 weeks for app testing by participants, 20 minutes for posttest questionnaires, and 20 minutes for semistructured interviews.

### Ethical Considerations

All data were collected in Italian and pseudonymized (deidentified) with participants’ informed consent. Confidential audio recordings of semistructured interviews were used for data analysis, and participants were identified only by numeric codes. At the study’s conclusion, participants can request the research outcomes from the research manager. Participants did not receive any compensation. This study was approved by the University of Padua Ethics Committee of Psychological Research on August 1, 2023 (reference 238-b).

### Data Analysis

Data analysis for the quantitative results of the semantic differential tool and uMARS questionnaires was conducted using JASP and R (R Foundation for Statistical Computing) [42,43]. Due to the small sample size, nonparametric tests were used [44]. When applicable, the Wilcoxon signed rank test (*W*) was used as a nonparametric alternative to the 1-sample 2-tailed *t* test [44-46], and the rank-biserial correlation (*r*) was reported to indicate the strength of association along with its corresponding 95% CI [46]. All analysis results were considered significant with a critical *P* value set at .05.

The data collected during the interviews were analyzed using a qualitative method. A thematic analysis was conducted [47], organizing the themes into tables based on different contexts and participant types (eg, psychologists and clinicians). The responses were analyzed by grouping the most prominent themes emerging from the ad hoc initial topics (around which the questions were formulated) into subvariables to address thematic redundancy within the participant sample. For this analysis, the interviews were first recorded and conducted by one author, whereas transcription and analysis were conducted by 2 other authors. Consensus on thematic relevance and redundancy was reached when both authors agreed; in case of disagreement, a third author was consulted to achieve a two-thirds majority. Finally, a comprehensive report was created highlighting the main findings with references to the specific groups where applicable.

## Results

### Phase 1: Iterative Development and Low-Fidelity Prototypes

**Figure 5:**
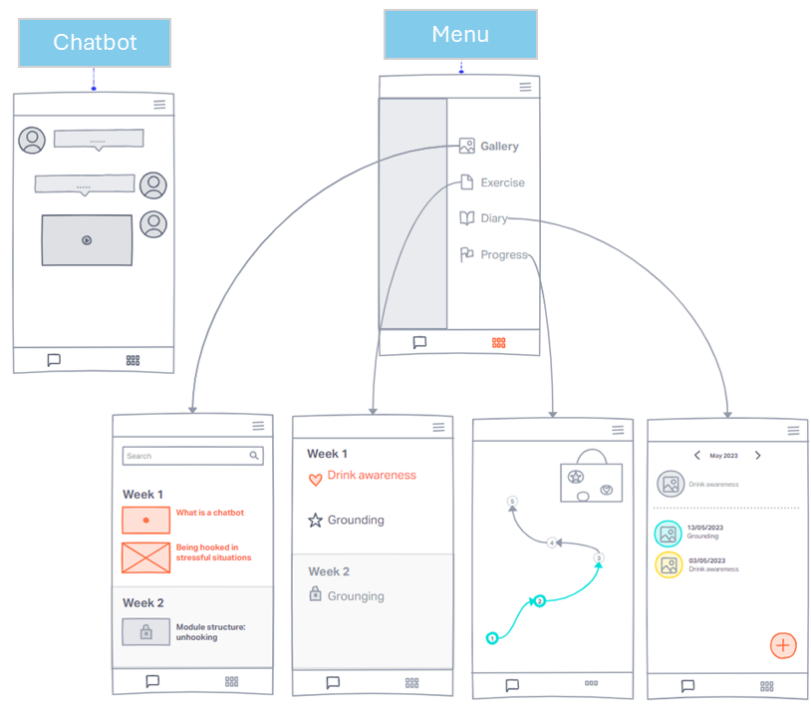
Low-fidelity prototype version of the ALBA app.

Specifically, the app was designed to include 5 sections: *Chatbot*, *Exercises*, *Diary*, *Gallery*, and *Progress*. The first low-fidelity prototype version of the app was developed (Figure 5).

#### Functions of App Sections

ALBA assigns homework during the session delivery that the users should carry out during the following days, so the app *Diary* section supports self-monitoring of exercise progress and completeness of sessions, reinforcing users’ empowerment. The completion of exercises is monitored in the evenings before the next session. All the exercises, divided week by week, are shown in the *Exercise* section. The *Gallery* page features all the multimedia material delivered by ALBA, such as educational videos, images, and intervention introductions. In addition, the app features a section called *Progress*, which provides users with an overview of their “journey” toward increased well-being. Using the journey metaphor, users can see the sticker badges earned by practicing exercises between sessions appearing on their suitcase illustration. These badges serve as long-term positive reinforcement and gratification for their efforts. Users receive badges for completion of exercises, and this is another way in which they can track their progress. Again, this gamification approach was selected to further reinforce empowerment and the sense of self-efficacy of the user.

#### Results of the Interaction With the Low-Fidelity Prototype

Regarding the interaction with the low-fidelity prototype in the co-design phase, 2 main themes emerged.

#### Communication

First, it was possible to review the communication style through role-play. To make the protocol more realistic, some parts of the dialogue were revised. The changes were made from a grammatical and syntactic point of view to make the text more fluid when reading. The changes also took empathy into account. This attention creates a feeling of trust in the relationship with ALBA. Through empathic communication, the person can feel in a safe space within which they are reassured and protected. For example, after these changes, ALBA provides the option to skip a particularly sensitive answer. It also lets the user find a calm place before doing audio exercises, and moreover, it gives the possibility to review the concepts of the previous session.

#### Duration

A second critical result that emerged from the specialists who tested the low-fidelity prototype was allowing the user to divide the session into 2 mini sessions. Given that the time needed to complete a single session is approximately 40 minutes, which is generally considered an excessive time to dedicate to interaction with an app. For this reason, appropriate changes were made to the dialogue to provide a partial and optional closure after 20 minutes followed by a gradual resumption of the session on the subsequent day. Thus, to support adherence, the session can be divided into 2 mini sessions, preventing user fatigue and improving the usability of both the chatbot and the app itself.

#### Other Improvements

At the same time, writing errors were corrected, and the remaining parts of the original protocols designed for groups were adapted to the individual intervention. Mainly, attention was placed on the translation from group to individual gratitude exercises, the final exercise of each session.

### Phase 2: Iterative Development and High-Fidelity Prototypes

#### Overview

**Figure 6:**
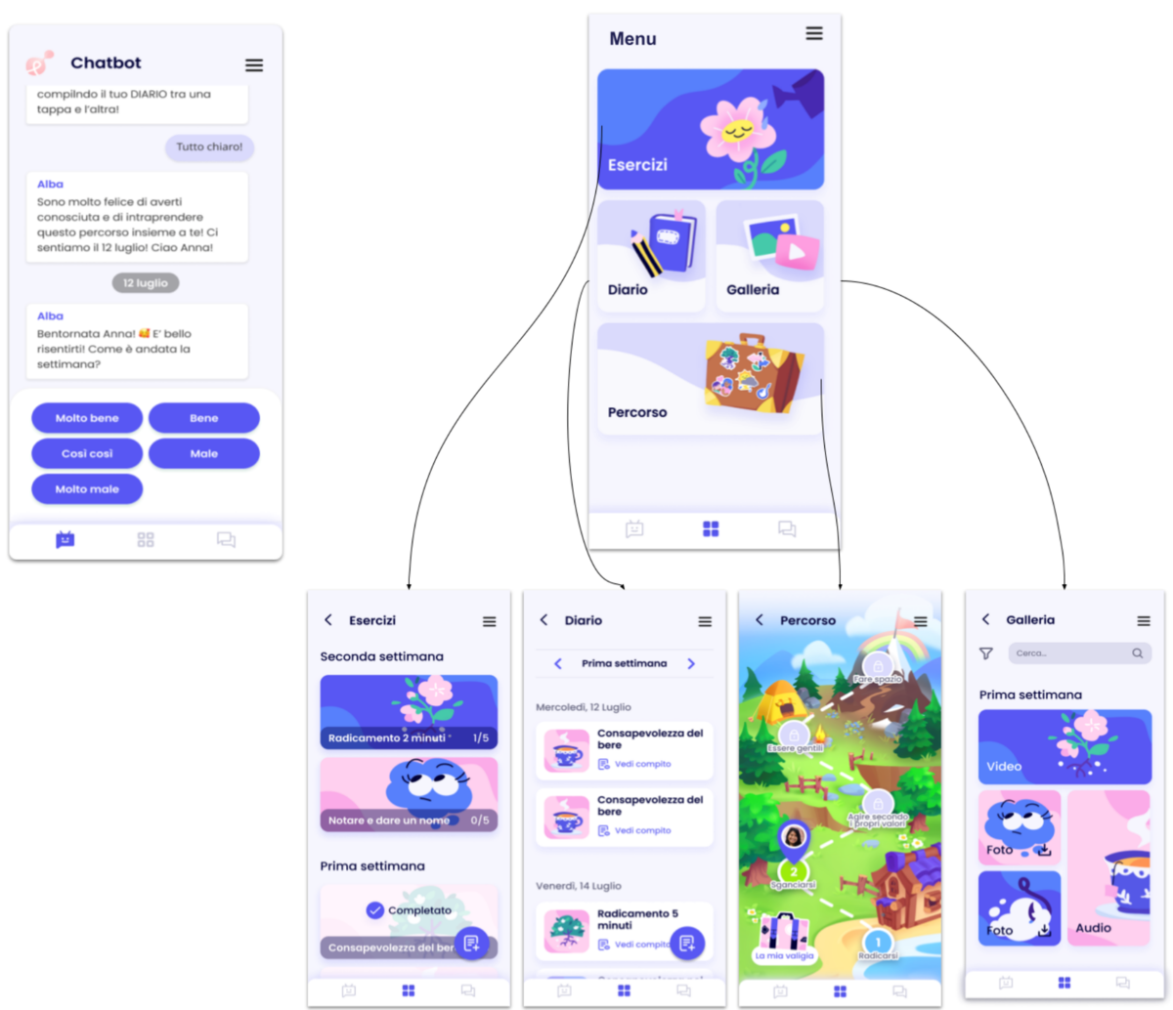
More realistic mock-ups of the ALBA app with its different sections.

To better understand the app and the entire intervention, more realistic mock-ups were created (Figure 6) after minor adjustments on the basis of the information gathered. This allowed users to better evaluate the app in its final appearance.

In total, 28 individuals (n=4, 14% men and n=24, 86% women) participated in the pilot evaluation; the average age was 41.39 (SD 11.66; range 28-72) years. The average number of schooling years of the sample was 18.98 (SD 2.88; range 13-23).

#### Quantitative Results

##### Semantic Differential Tool

In this case, using a semantic differential–based questionnaire also facilitated the observation of the respondents’ average positioning with respect to the 3 macrovariables under investigation. In particular, concerning the subvariables, several significant results emerged from the Wilcoxon analysis, as reported in Table 2. The graphical representation of mean values derived from the semantic differential tool for each item is reported in Multimedia Appendix 3. As seen in this figure, a tendency toward the positive semantic pole (right pole) emerged in the feedback of this group of participants.

**Table 2:** Results of the semantic differential tool (N=28)^a^.

	Participants, n (%)	Values, mean (SD)	Values, median (range)	*W*	*P* value	*r* (95% CI)
Empathy and listening	25 (89)	3.55 (0.67)	3.60 (2.40-5.00)	213.50	<.001	0.85 (0.64 to 0.94)
Smoothness and fluidity	28 (100)	3.89 (0.69)	4.00 (2.00-5.00)	266.00	<.001	0.93 (0.83 to 0.97)
Chatbot interaction	28 (100)	3.72 (0.55)	3.75 (2.50-4.50)	271.50	<.001	0.97 (0.92 to 0.99)
Lexicon	28 (100)	4.41 (0.61)	4.50 (2.50-5.00)	404.00	<.001	0.99 (0.98 to 1.00)
Session structure	28 (100)	2.95 (0.63)	3.00 (1.00-4.00)	64.50	.87	−0.05 (−0.54 to 0.47)
Audio tracks	25 (89)	3.97 (0.44)	4.00 (3.00-5.00)	300.00	<.001	1.00 (1.00 to 1.00)
Infographics and videos	25 (89)	4.08 (0.50)	4.00 (3.00-5.00)	300.00	<.001	1.00 (1.00 to 1.00)

^a^For the Wilcoxon test, the effect size is given by the matched rank-biserial correlation.

The results indicate that participants generally responded positively across various subvariables. Significant positive responses were observed for empathy and listening, smoothness and fluidity, chatbot interaction, lexicon, audio tracks, and infographics and videos. The session structure subvariable did not show a significant positive trend, indicating a neutral or mixed response. The effect sizes ranged from moderate to large, suggesting varying degrees of impact for the different subvariables under investigation.

##### uMARS Results

The uMARS evaluated the respondents’ average positioning in 4 key dimensions. Detailed results of the Wilcoxon tests are summarized in Table 3.

**Table 3:** Results of the User Version of the Mobile Application Rating Scale (N=28)^a^.

	Participants, n (%)	Values, mean (SD)	Values, median (range)	*W*	*P* value	*r* (95% CI)
Engagement	28 (100)	3.55 (0.43)	3.60 (3.00-4.60)	300.00	<.001	1.00 (1.00-1.00)
Functionality	28 (100)	4.16 (0.51)	4.25 (3.00-5-00)	378.00	<.001	1.00 (1.00-1.00)
Esthetics	28 (100)	3.86 (0.49)	4.00 (3.00-4.67)	300.00	<.001	1.00 (1.00-1.00)
Information	28 (100)	4.20 (0.52)	4.25 (2.50-5.00)	405.00	<.001	1.00 (0.99-1.00)
Subjective items	28 (100)	3.20 (0.50)	3.25 (2.25-4.00)	233.50	.06	0.44 (0.02-0.72)
Perceived impact	28 (100)	3.66 (0.60)	3.83 (2.67-4.50)	264.00	<.001	0.91 (0.79-0.97)

^a^For the Wilcoxon test, the effect size is given by the matched rank-biserial correlation.

The results indicate consistently high ratings across all dimensions of the uMARS. Significant positive responses were noted for *Engagement*, *Functionality*, *Esthetics*, *Information*, and *Perceived Impact*. Effect sizes were uniformly high, particularly for the Wilcoxon tests, indicating a strong positive skew in user perceptions for each dimension evaluated. However, items such as “Customization” and “Interactivity” did not show significant positive trends in the *Engagement* scale, with “Customization” even indicating a negative effect size, suggesting variability or mixed responses from users.

Considering the *Subjective Items* scale singularly (which did not show a significant trend), the items “Would you recommend” (median 4.00; SD 0.62; *P*<.001; effect size=1.00) and “Overall rating” (median 4.00; SD 0.52; *P*<.001; effect size=1.00) received a strong positive response. This suggests that most users would recommend the app to others, with a robust positive effect size indicating widespread satisfaction with the app’s performance, utility, and perceived value. However, for the item “How many times” (median 3.00; SD 0.77; *P*=.64; effect size=0.13), responses were mixed regarding the frequency of app use, with no significant trend emerging. This indicates that users were likely to use the app on average 3 to 10 times in the following year. Moreover, for the item “Would you pay” (median 2.00; SD 0.72; *P*<.001; effect size=−1.00), there was a significant negative response, indicating that users were generally unwilling to pay for the app. The strong negative effect size underscores a consistent reluctance to incur costs for app use.

Regarding the relevant *Perceived Impact* scale of the uMARS, items such as “Awareness” (median 4.00; SD 0.74; *P*<.001; effect size=0.24), “Knowledge” (median 4.00; SD 0.79; *P*<.001; effect size=0.26), “Attitudes” (median 3.50; SD 0.73; *P*=.02; effect size=0.26), “Intention to Change” (median 4.00; SD 0.69; *P*=.001; effect size=0.26), and “Help Seeking” (median 4.00; SD 1.09; *P*=.003; effect size=0.23) were rated positively. This indicates that the app positively impacted users, although the effect sizes were lower compared with those of other items, suggesting a moderate consensus and some variability in responses. Conversely, the “Behavior Change” item result (median 4.00; SD 0.64; *P*<.001; effect size=0.88) highlights that the app had a significant positive impact on users, with a high effect size indicating strong agreement among users on the app’s effectiveness in promoting behavioral modifications.

**Figure 7:**
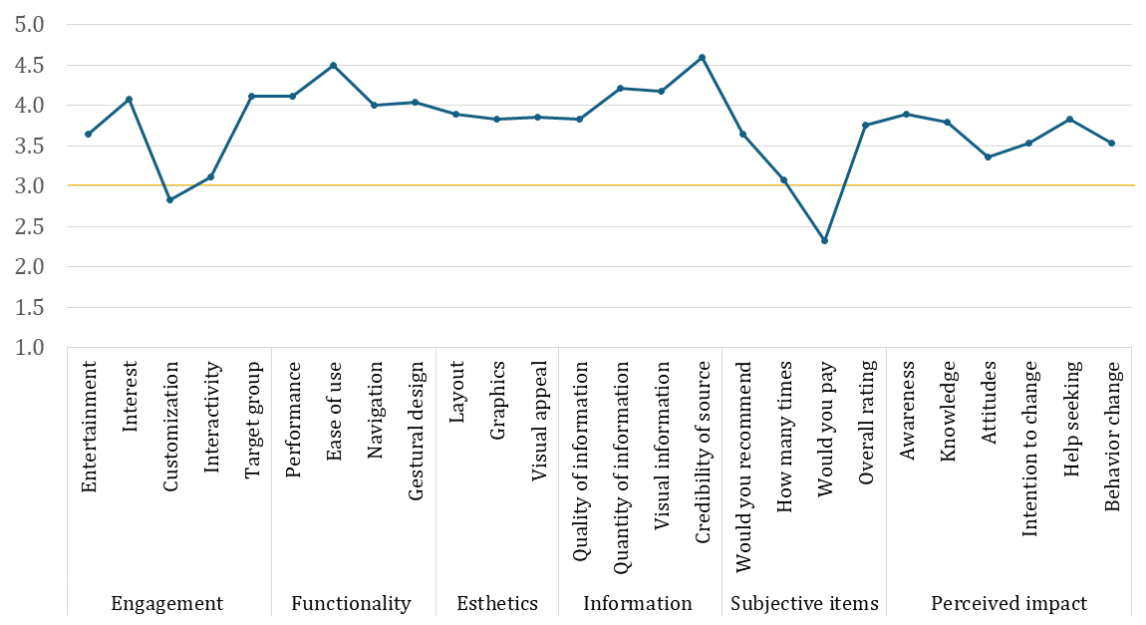
Graphical distribution of User Version of the Mobile Application Rating Scale item scores.

The mean and statistical significance for every item are reported in Multimedia Appendix 4. Figure 7 shows the graphical distribution of item scores.

#### Qualitative Results: Semistructured Interviews

One psychologist conducted the qualitative interview to gather additional information. Interviews were conducted and analyzed according to the identified thematic variables.

##### Set 1: Interaction

The interaction with the chatbot was generally well received. The clinicians appreciated the good overall interaction, pointing out the effectiveness of the videos and images as well as the presence of multiple response options. However, they found it difficult to go back to previous answers, noting repetitiveness and lack of novelty in the content. The communication experts praised the fluidity of the interaction and particularly appreciated the personality of the app but criticized the excessively fragmented design of the messages.

The SH+ expert highlighted the confidence given by the chatbot, which calls the user by name and provides relevant answers, but it was not clear when user responses could be expressed in free text (chatting and not pressing the default buttons) and expressed a preference for the human figure in the videos. The usability experts appreciated the guidance and psychoeducational support offered by the chatbot but pointed out the 1-way interaction and the excessive number of messages in a row.

Among the features preferred by those who tested the app were videos with exercises, the personality of the app, heterogeneous content, and images with goals and values. However, among the elements to be improved were some aspects of the videos. In particular, the psychologists pointed out that professional voices could greatly improve the effectiveness of the videos, which were nevertheless appreciated for their content. The SH+ expert, on the other hand, found the videos and audio a little too slow and monotonous but greatly appreciated the chatbot’s confidence and relevant answers. Finally, the usability experts found the videos and graphics useful but criticized the excessive length of the messages.

There was some discord regarding the possibility of correcting oneself and going back. In fact, the SH+ expert and psychologists considered it very useful and suggested that it should be included, whereas the communication experts did not find it particularly useful to go back. The usability experts did not find frequent errors, and the psychologists considered the consequences of errors to be not serious. On the other hand, the target group appreciated the practical examples provided during the sessions, which were considered important and highly functional to the objectives of the intervention. The variety of response options was also positively evaluated. However, it was suggested to reduce the length of the videos and messages as shorter and more focused content was deemed to improve the overall experience.

##### Set 2: Communication Mode

Concerning clarity and thoroughness, all categories of experts appreciated the possibility to better investigate concepts through examples and videos. In particular, the communication experts emphasized the clarity of the content, although they suggested using more concise answers. The same suggestion was made by the SH+ expert, who found some dialogues too complex and articulate. The clinicians disagreed, instead pointing out the simple and accessible language.

The usability experts also noted that the repetition of information was positive, especially for weekly use, but found the audio and video a little too slow and monotonous. Similarly, the psychologists appreciated the repetition of videos to better explain concepts and suggested alternating text with images or videos to lighten the information load.

The language used by the chatbot was generally well received. The clinicians appreciated the simple language and short sentences, whereas the communication and usability experts praised the clear, appropriate, and friendly language. The psychologists and SH+ expert also found the language appropriate to the content and easy to understand. However, the SH+ expert considered the tone to be a little slow. It was suggested to keep the language simple but to consider more complexity and variety in the content using a more active and engaging tone.

Finally, the target group generally appreciated the appropriateness of the sentence length and the terminology used as the language was perceived as accessible and aligned with the needs of future users. In addition, the inclusion of emojis was seen as effective and realistic in conveying emotions. Users found these visual elements to be a helpful and engaging way to express their feelings. All categories of experts appreciated and found useful the visual mode of emojis, which was also considered effective by the usability experts. However, it was suggested to offer both emojis and words as a response option to accommodate different preferences. However, one area for improvement pointed out by the target group was the length of the written messages. Some users reported that, sometimes, the messages were too long and could become heavy to read.

##### Set 3: Involvement and Consistency

User involvement and consistency were strengths for some groups, whereas others experienced difficulties. The clinicians found the tone positive and encouraging and the communication fast. The SH+ expert found the communication engaging, whereas the psychologists found good engagement in the videos. However, the clinicians found it difficult to feel engaged without a physical person, whereas the usability experts found the interaction similar to reading a book, needing more interactivity and exchange. For these reasons, it was suggested that the level of personalization and interaction be improved to increase engagement.

The customization of the chatbot was considered good but with room for improvement. The clinicians appreciated the personalization through the use of the user’s name and the space for personal choices but noted standard answers for all and the lack of specific personalization elements. The SH+ expert gave a score of 8/10 to personalization, whereas the psychologists noted that the answers were standard. It was suggested to offer the possibility to add photos and avatars and improve the customization of answers. The target group found the content engaging and motivating to continue, with good personalization using names. However, they noted that it became less engaging in the long term, with repetition feeling lengthy and tedious and the exercises sometimes being monotonous.

##### Set 4: General Questions

The communication experts noted that the timing should be personalized according to individual circumstances, emphasizing that some users might benefit more from reminders and progress tracking. Indeed, target users indicated that the chatbot could be helpful during times of high stress and change, such as during chemotherapy or radiation and after childbirth, but less useful immediately after diagnosis or in early pregnancy. In contrast, the clinicians suggested that the ideal time for chatbot use in oncology is during and after chemotherapy or radiation treatment, whereas during pregnancy, it is in the first and second trimesters. In addition, the SH+ expert expressed that the chatbot was generally useful but highlighted the need for it to be adaptable across different stages of treatment and pregnancy. The psychologists found consistency with the ACT model and good care in the messages but reported that negative responses were not always considered.

##### Set 5: Technical Implementation

Reminders and notifications were considered useful for maintaining consistency. However, it was emphasized that reminders should not be too intrusive. The communication experts emphasized the importance of sending reminders in a nondisruptive way, whereas the SH+ expert stressed the need for reminders to maintain consistency. The usability experts agreed with the clinicians but suggested integrating reminders and immediate feedback to enhance user engagement. In particular, the target group appreciated the overall coherence of the content with the topic addressed and the usefulness of reminders to maintain consistency in use. However, they also reported usability issues, especially when too many messages were received in sequence, which made them difficult to manage and slowed down interaction with the chatbot.

Concerning gamification aspects, the preference for gradual coloring of the stickers was expressed by most groups. The clinicians suggested that gradual coloring gives the idea of progress, whereas the communication experts preferred immediate gratification. The SH+ expert indicated a preference for receiving the sticker at the end, whereas the usability experts suggested giving the reward immediately to maintain engagement. The target group preferred a gradual color progression of the stickers as they completed the exercises. Indeed, this approach helps visualize progress, providing them satisfaction and motivation. At the same time, a lot of users also expressed the desire for immediate rewards and feedback to maintain engagement. Indeed, the use of pop-up positive feedback provides instant gratification and motivation.

##### Set 6: Content Adherence

The SH+ expert reported that, on a scale from 1 to 5, our intervention adhered at a level of 4 to the original intervention. In terms of content, the app takes them up and is coherent, yet it is very innovative in terms of the ways it is delivered.

Table 4 briefly highlights key positive and negative aspects derived from the user interviews.

**Table 4:** Main positive and negative aspects that emerged from the user interviews.

Variable and subvariable			Positive aspects		Negative aspects (and suggestions)
**Set 1: interaction**					
	General+alternatives for answers	Positive interaction (n=5)^a^; multiple options for answers (n=10)		Limited alternatives in some cases (n=5); fatigue in going back to previous answers (n=2)	
	Best and worst features	Confident and personalized chatbot; interactive videos and images (n=9)		Long and sometimes monotonous videos (n=4)	
	Going back for mistakes	—^b^		Need for option to correct mistakes (n=13)	
**Set 2: communication mode**					
	Clarity	Clear language (n=10); suitable for all users (n=2)		Sometimes overly simplistic and repetitive (n=1)	
	Emojis	Visual and easy to express emotions (n=8)		Offer both emojis and words for responses to cater to different preferences (n=6)	
	Length of and terms used in messages	Terms appropriate to the content (n=13)		Messages can be too lengthy (n=5)	
**Set 3: involvement and consistency**					
	Engagement	Positive and encouraging tone (n=1)		Improve personalization (n=7)	
	Personalization	Use of user’s name (n=4); space for personal input (n=3)		Lacks deeper personalization (n=6)	
**Set 4: general questions**					
	Concerns and criticism	Reflective questions after exercises (n=1)		Even if the user can read the message, they can still decide not to proceed (n=1)	
	Ideal time	Oncology: during (n=11) and after (n=8) treatment; pregnancy: second trimester (n=10) and after (n=12) childbirth		Not ideal immediately after diagnosis (n=5) or in early pregnancy (n=4)	
**Set 5: technical implementation**					
	Reminders	Helpful for maintaining consistency (n=22)		Ensure that reminders are supportive and not overwhelming (n=5)	
	Pop-up positive feedback	Provides instant gratification and motivation (n=17)		Offer immediate feedback but consider a weekly summary for sustained engagement (n=1)	
	Progress in stickers	Gradual coloring indicates progress and provides satisfaction (n=19)		—	
**Set 6: content adherence**					
	Adherence to SH+^c^ protocol	Generally aligns well with SH+ protocol (n=1)		Repetitive content (n=3)	

^a^Numbers in parentheses indicate the response frequency.

^b^No statements.

^c^SH+: Self-Help Plus.

## Discussion

### Principal Findings

This study evaluated the adaptation of the WHO SH+ intervention for stress management. The first step involved developing the SH+ protocol, which was implemented through a mobile app with the support of the interactive ALBA chatbot. ALBA guides users through the 5-week program, corresponding to the 5 SH+ sessions, and facilitates navigation through various app sections. Notably, this adaptation introduced several innovations: the intervention was designed to further reinforce interaction and feedback to foster empowerment and self-efficacy, and it was tailored to female users, explicitly targeting 2 populations of interest in our study—pregnant women and women diagnosed with breast cancer.

The ORBIT and the CeHRes comprehensive road map approaches were adopted to evaluate the app, starting with a preliminary phase focused on refining the dialogues and low-fidelity mock-ups of the app’s sections. This initial phase was crucial for developing coherent, accurate, and engaging dialogues and ensuring a reliable adaptation of the original SH+ content. Given that the protocol had already been validated, the chatbot’s structured and standardized dialogues allowed for effective transmission of the proven content without the risk of artificial hallucinations, undertaken risks, or biases, which can occur with more advanced chatbot models based on large language models [48]. Giving value to the methodology adopted, regarding content adherence, the SH+ expert rated the intervention’s adherence to the original at 4 out of 5, appreciating the innovative delivery methods while the core content was maintained. However, an explicit limitation of our chatbot emerged—the lack of flexibility in personalizing responses and interactions, as highlighted by participants in phase 2. Indeed, it is worth noting that psychologists found consistency with the ACT model but noted that negative responses were not always considered by ALBA. This rigidity presents a double-edged sword—while it ensures the psychological rigor of the initial intervention, it also reduces the flexibility of personalized response options [49].

Additional relevant findings from the low-fidelity prototype review pertain to the app’s organization, featuring a well-defined structure in sections evaluated during the second phase regarding usability. The integration of gamification and feedback aspects based on user progress monitoring to maintain engagement and immediate or delayed reinforcement from classic behaviorism to sustain motivation was also significant. The app provided customization in session management by proposing interruptions and clarification moments for the presented content, which users could accept or decline.

From the second phase of this study, further essential results emerged that will guide the iterative development of the app. Valuable insights were gathered by involving various stakeholder groups identified from both the pregnancy and oncology contexts. Both expert groups and target users, the final app users, provided quantitative feedback through the semantic differential tool and uMARS questionnaires and qualitative feedback through semistructured interviews. The evaluation of dialogues and mock-ups, following modifications from phase 1, confirmed a generally positive assessment of the app and the ALBA chatbot.

In particular, the semantic differential tool results indicated that ALBA’s communication was empathetic and fluid, and the interaction with users was deemed appropriate and acceptable. In addition, the interview reports highlighted that the interaction with the chatbot was generally well received, although criticisms included difficulties in navigating back to previous answers, repetitiveness, lack of novelty, and fragmented message design. Specific feedback highlighted the chatbot’s confidence, relevant responses, and psychoeducational support while suggesting improvements such as professional voices for videos and reducing video and message length. Overall, both the target group and experts acknowledged the strengths of the chatbot’s interaction, particularly its engaging multimedia elements and responsive nature. However, their perspectives diverged significantly on several key aspects. The target group expressed a desire for more innovative content and a reduction in repetitiveness, emphasizing that fresh and varied interactions would enhance their experience. In contrast, the experts, particularly the communication and usability specialists, focused on the fragmented design of the messages and the overwhelming volume of content, suggesting that a more cohesive and streamlined message structure would improve interactive usability. Indeed, user satisfaction significantly improves when chatbots provide quick, relevant, and friendly responses [50]. This capability reduces wait times and enhances the perception of service efficiency. Moreover, positive interactions with chatbots not only increase short-term satisfaction but also contribute to long-term user loyalty as users with positive experiences are more likely to return and use the service again, thereby strengthening their relationship with the app [51].

Regarding the communication modality, from the semantic differential tool emerged that the language was clear and understandable. Experts valued the clarity and thoroughness of using examples and videos to investigate concepts, suggesting more concise answers and alternating text with images or videos. Thus, multimedia content, such as images, videos, and audio provided by the chatbot, was also positively recognized both in the semantic differential tool and interview outputs. While the chatbot’s language was also praised for simplicity and clarity in the interviews, some found the tone slow and recommended a more active tone, and the use of emojis was well received, with a recommendation to offer both emoji and word response options; the target group found the terms satisfactory but suggested reducing message length. In comparing the perspectives of experts and the target group on communication mode, notable differences emerged. The experts emphasized the importance of clarity and the effective use of various communication modalities, highlighting the need for dynamic and engaging delivery. In contrast, the target group appreciated the overall clarity and accessibility of the language used but expressed concerns about the length of certain messages. While the experts focused on enhancing different communication modalities, the target group prioritized brevity and engaging content. This divergence in focus highlights specific areas for improvement, indicating that refining the chatbot’s communication strategies could enhance user satisfaction and overall effectiveness.

Overall, the session structure was considered appropriately lengthy and moderately light in content according to the semantic differential tool results. The importance of using inclusive and comprehensible language in chatbots is increasingly recognized in academic literature, emphasizing how this can enhance user experience and engagement. Studies show that chatbots that use such language can significantly improve user satisfaction and accessibility, making interactions more effective and welcoming for a diverse audience [52,53].

Regarding the uMARS standardized questionnaire, equally encouraging results were obtained for the engagement, functionality, esthetics, and information variables. However, improvements are needed for customization and interactivity, which could have received a more clearly positive rating. In accordance with this, in the interviews, the usability experts appreciated the guidance and psychoeducational support from the chatbot but noted a 1-way interaction and too many consecutive messages. Indeed, the experts, including the clinicians, SH+ specialist, and psychologists, noted in the interviews the difficulties related to the lack of physical presence and the static nature of the interaction, which felt akin to reading a book. They emphasized the need for enhanced personalization and interaction to foster deeper engagement, with suggestions to include features such as photos and avatars. Additional insights on engagement from interview responses were mixed. Although personalization through the use of the user’s name was appreciated by the target group, there was a need for more specific customization—while the content was initially engaging and motivating for the target group, it became less engaging over time, leading to suggestions for reducing repetition and monotonous exercises. This divergence highlights the experts’ focus on improving interaction dynamics and customization, whereas the target group prioritized sustained engagement and the need for variety to maintain interest over time.

Enhancements are expected in these areas by better integrating the *Diary* and *Exercise* sections with the chatbot’s messages regarding weekly exercise management once the app is fully implemented. In addition, features such as reminders and feedback, which were not available to testers, are expected to improve the perception of app personalization. The literature emphasizes the effectiveness of engagement strategies and reminders in improving user interaction with chatbots. Recent research shows that chatbots that use personalized engagement techniques and timely reminders can significantly enhance user commitment over time and adherence to recommended actions, leading to better outcomes and increased user satisfaction [54].

Additional information from the interviews provides insights about future technical implementation. Both the experts and the target group recognized the utility of reminders and notifications but mentioned that they should not be intrusive, with preferences for nondisruptive reminders and maintaining consistency. Moreover, most groups preferred a gradual coloring of stickers to indicate progress, providing satisfaction and motivation, although the target group also expressed a desire for immediate rewards, indicating a nuanced understanding of how to balance engagement and consistency. The app’s ease of use and the high perceived credibility of the source were 2 very positive aspects highlighted. Both results will be further investigated in future feasibility studies using standardized tools to measure app usability [55] and user trustworthiness [56].

A good overall rating emerged from the uMARS items regarding subjective impact and perception, with behavior-change and help-seeking initiatives aligning with the intervention principles. These results are promising for the app’s effective use but should be considered with the potential bias from psychological experts who favor such interventions. In addition, participants indicated a reluctance to pay for the app hypothetically and suggested that they would use it 3 to 10 times per year. While the first point may seem moderate, the app will be provided for free by the health care system, and some willingness to pay adds value. The second point warrants further investigation as the app is designed for continuous use over 5 weeks and it is unclear whether further use throughout the year implies single accesses or restarting of the intervention. The interviews indicated that the app could be proposed to target women at various stages of breast cancer care or before or after childbirth in other contexts of interest. The clinicians suggested using the chatbot during and after chemotherapy or radiation for oncology patients and during the first and second trimesters for pregnant women. Similar ideas emerged from the target group. They indicated that the chatbot could be helpful during chemotherapy or radiation and after childbirth and, in contrast, less useful immediately after diagnosis or in early pregnancy. This flexibility is due to the different triggers and timings of stress-related issues in both contexts [57,58].

### Strengths, Limitations, and Future Directions

This study highlights several strengths, limitations, and future directions for the app. Among the strengths, the chatbot’s structured and standardized dialogues ensured an effective delivery of the validated content, and the empathetic dialogues and integration of gamification and feedback mechanisms were positively received by participants. In addition, the app’s ease of use and high perceived credibility of the contents coming from a validated WHO protocol were crucial for user adoption. However, notable limitations include the chatbot’s rigidity in personalizing responses and interactions and some repetitive content.

There was also a clear need for more specific customization options and improving answer personalization. While initially engaging, the content became less motivating over time, prompting suggestions to reduce repetition and monotonous exercises.

Future directions involve enhancing the chatbot’s flexibility and personalization by incorporating user-specific elements. Implementing reminders and feedback mechanisms is also expected to improve personalization perception. Furthermore, conducting future feasibility studies using standardized tools to measure app usability and user trustworthiness will be essential. Exploring the frequency and context of app use over a year will help better understand user needs and improve continuous engagement effectiveness in pregnancy and oncological contexts.

### Conclusions

This research evaluated the adaptation of the SH+ intervention for stress management through a mobile app guided by the ALBA chatbot. The implementation of the protocol tailored for pregnant women and women with a breast cancer diagnosis showcased several innovations, including interactive elements, gamification, and personalized feedback mechanisms. Using the ORBIT and CeHRes methodologies, this study validated the structured dialogues of the chatbot for effective content transmission while acknowledging limitations such as rigidity in personalization. Despite these challenges, the app’s organization, user-friendly interface, and perceived credibility were notable strengths identified through participant feedback. Moving forward, addressing customization shortcomings, enhancing engagement strategies, and conducting further usability studies will be critical to refining the app’s effectiveness and user satisfaction across diverse health care contexts.

## Appendix

Multimedia Appendix 1 List of variables, subvariables, and adjectives investigated using the semantic differential tool.

Multimedia Appendix 2 Questions posed to participants attending the interview evaluation.

Multimedia Appendix 3 Graphical representation of mean values derived from the semantic differential tool.

Multimedia Appendix 4 Wilcoxon signed ranked test analysis of the User Version of the Mobile Application Rating Scale items.

## Abbreviations

ACT: acceptance and commitment therapy
CeHRes: Center for eHealth Research and Disease Management
mHealth: mobile health
ORBIT: Obesity-Related Behavioral Intervention Trials
SH+: Self-Help Plus
uMARS: User Version of the Mobile Application Rating Scale
WHO: World Health Organization
