# Supplementary material for: A Chatbot-Based Version of the World Health Organization–Validated Self-Help Plus Intervention for Stress Management: Co-Design and Usability Testing
Source: JMIR Hum Factors. 2024 Oct 18;11:e64614. doi: 10.2196/64614 (PMC11530720; doi:10.2196/64614)
Supplement: Multimedia Appendix 1 [file humanfactors_v11i1e64614_app1.docx]

## **Multimedia Appendix 1**

Table S1. List of variables, subvariables, and adjectives investigated using the semantic differential tool.

| **Variables** | **Sub-Variables** | **Adjectives** |
| --- | --- | --- |
| Communication | Empathy and listening  Smoothness and fluidity  Chatbot interaction  Lexicon | judgmental - welcoming  passive listening - active listening alarming - reassuring  indifferent - sensitive  cold - warm  non-flowing - flowing  boring - engaging  inefficient - efficient  slow - fast  pressing - adequate  abstruse - understandable  technical - common |
| Session structure | Interaction length | long - short  demanding - light |
| Materials | Audio tracks  Infographics and videos | unpleasant - pleasant  stressful - relaxing  in the way - supportive  useless - functional  unimaginative - creative  hindering - supportive  useless - functional |
