# Supplementary material for: A Chatbot-Based Version of the World Health Organization–Validated Self-Help Plus Intervention for Stress Management: Co-Design and Usability Testing
Source: JMIR Hum Factors. 2024 Oct 18;11:e64614. doi: 10.2196/64614 (PMC11530720; doi:10.2196/64614)
Supplement: Multimedia Appendix 2 [file humanfactors_v11i1e64614_app2.docx]

## **Multimedia Appendix 2**

Table S1. Questions posed to participants attending the interview evaluation.

| **Topic investigated** | **Questions** |
| --- | --- |
| Set 1: Interaction | 1.1 How did you feel about the interaction with ALBA? Were there enough alternatives among the replies to ALBA?  1.2 What was the feature of the interaction that you liked the most? And the one you liked the least?  1.3 Have you ever made the mistake of clicking the answer button on a question? Have you wished you could have gone back? |
| Set 2: Communication mode | 2.1 If something was not clear to you at first, do you find there is then a way to investigate the topic further?  2.2 Did you like answering the question "How are you?" from session 2 with emojis, or did you prefer to answer with words?  2.3 Is the mode of communication (length of sentences, terms used) appropriate for the content? |
| Set 3: Involvement and constancy | 3.1 Was the communication with the chatbot engaging? Did it entice you to get involved and be consistent in activities?  3.2 Overall, was the intervention personalized for you? Give a level of personalization from 0 to 10. |
| Set 4: General questions | 4.1 Do you have any concerns? Do you have any criticism?  ONLY CLINICIANS AND TARGETS OF THE SPECIFIC CONTEXT 4.2 When would be the ideal time to take this intervention for a pregnant woman or a breast cancer patient? |
| Set 5: Technical implementation | 5.1 Would reminders help you be more consistent in doing exercises or completing dialogues?  5.2 Would you like to receive the pop-up message that you reached the goal as soon as you did? Or would you prefer to receive it at the end of the week?  5.3 Would you like to receive the sticker in black and white for completing the dialogue and then coloured for completing the exercises? Or would you prefer it to be gradually coloured as you proceeded to complete the exercises? |
| Set 6: Content adherence | ONLY SH+ EXPERT 6.1 Did the intervention comply with the original SH+ protocol? On a scale from 1 to 5? |
