# Supplementary figures and images for: A Chatbot-Based Version of the World Health Organization–Validated Self-Help Plus Intervention for Stress Management: Co-Design and Usability Testing
Source: JMIR Hum Factors. 2024 Oct 18;11:e64614. doi: 10.2196/64614 (PMC11530720; doi:10.2196/64614)

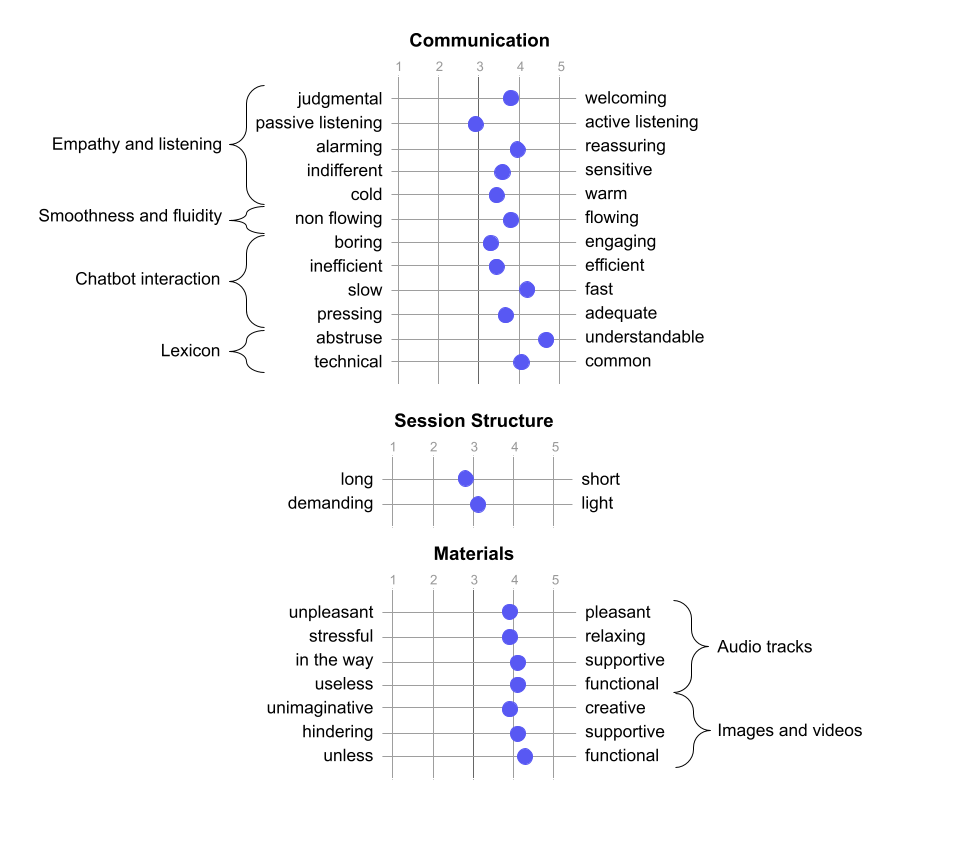

Supplement: Multimedia Appendix 3 [file humanfactors_v11i1e64614_app3.png]
