# Supplementary material for: A Chatbot-Based Version of the World Health Organization–Validated Self-Help Plus Intervention for Stress Management: Co-Design and Usability Testing
Source: JMIR Hum Factors. 2024 Oct 18;11:e64614. doi: 10.2196/64614 (PMC11530720; doi:10.2196/64614)
Supplement: Multimedia Appendix 4 [file humanfactors_v11i1e64614_app4.docx]

## **Multimedia Appendix 4**

Table S1. Wilcoxon signed ranked test analysis of the User Version of the Mobile Application Rating Scale items.

| ***uMARS*** | | | | | | | | ***95% CI for Effect Size*** | |
| --- | --- | --- | --- | --- | --- | --- | --- | --- | --- |
|  | ***N*** | ***M*** | ***Mdn*** | ***SD*** | ***W*** | ***P*** | ***r*** | ***Lower*** | ***Upper*** |
| **Entertainment** | 28 | 3.64 | 4.00 | 0.56 | 199.50 | <.001 | 0.90 | 0.75 | 0.96 |
| **Interest** | 28 | 4.07 | 4.00 | 0.54 | 325.00 | <.001 | 1.00 | 1.00 | 1.00 |
| **Customization** | 28 | 2.82 | 3.00 | 0.86 | 42.00 | 0.30 | -0.29 | -0.70 | 0.27 |
| **Interactivity** | 28 | 3.11 | 3.00 | 0.74 | 72.00 | 0.46 | 0.20 | -0.36 | 0.65 |
| **Target group** | 28 | 4.11 | 4.00 | 0.79 | 231.00 | <.001 | 1.00 | 1.00 | 1.00 |
| **Performance** | 28 | 4.11 | 4.00 | 0.69 | 276.00 | <.001 | 1.00 | 1.00 | 1.00 |
| **Ease of use** | 28 | 4.50 | 5.00 | 0.64 | 351.00 | <.001 | 1.00 | 1.00 | 1.00 |
| **Navigation** | 28 | 4.00 | 4.00 | 0.54 | 300.00 | <.001 | 1.00 | 1.00 | 1.00 |
| **Gestural design** | 28 | 4.04 | 4.00 | 0.64 | 276.00 | <.001 | 1.00 | 1.00 | 1.00 |
| **Layout** | 28 | 3.89 | 4.00 | 0.74 | 190.00 | <.001 | 1.00 | 1.00 | 1.00 |
| **Graphics** | 28 | 3.82 | 4.00 | 0.72 | 171.00 | <.001 | 1.00 | 1.00 | 1.00 |
| **Visual appeal** | 28 | 3.86 | 4.00 | 0.53 | 253.00 | <.001 | 1.00 | 1.00 | 1.00 |
| **Quality of information** | 28 | 3.82 | 4.00 | 0.67 | 243.00 | <.001 | 0.92 | 0.81 | 0.97 |
| **Quantity of information** | 28 | 4.21 | 4.00 | 0.69 | 300.00 | <.001 | 1.00 | 1.00 | 1.00 |
| **Visual information** | 28 | 4.18 | 4.00 | 0.61 | 395.00 | <.001 | 0.95 | 0.88 | 0.98 |
| **Credibility of source** | 27 | 4.59 | 5.00 | 0.69 | 300.00 | <.001 | 1.00 | 1.00 | 1.00 |
| **Would you recommend** | 28 | 3.64 | 4.00 | 0.62 | 136.00 | <.001 | 1.00 | 1.00 | 1.00 |
| **How many times** | 28 | 3.07 | 3.00 | 0.77 | 76.50 | .64 | 0.13 | -0.41 | 0.59 |
| **Would you pay** | 28 | 2.32 | 2.00 | 0.72 | 0.00 | <.001 | -1.00 | -1.00 | -1.00 |
| **Overall rating** | 28 | 3.75 | 4.00 | 0.52 | 210.00 | <.001 | 1.00 | 1.00 | 1.00 |
| **Awareness** | 28 | 3.89 | 4.00 | 0.74 | 244.00 | <.001 | 0.24 | 0.83 | 0.92 |
| **Knowledge** | 28 | 3.79 | 4.00 | 0.79 | 182.50 | <.001 | 0.26 | 0.79 | 0.97 |
| **Attitudes** | 28 | 3.36 | 3.50 | 0.73 | 133.00 | .02 | 0.26 | 0.10 | 0.82 |
| **Intention to change** | 28 | 3.54 | 4.00 | 0.69 | 153.00 | .001 | 0.26 | 0.50 | 0.92 |
| **Help seeking** | 28 | 3.82 | 4.00 | 1.09 | 252.00 | .003 | 0.23 | 0.36 | 0.86 |
| **Behaviour change** | 28 | 3.54 | 4.00 | 0.64 | 128.00 | <.001 | 0.88 | 0.68 | 0.96 |
| Note. For the Wilcoxon test, effect size is given by the matched rank biserial correlation. | | | | | | | | | |
